# Relationship between Programmed Cell Death Protein Ligand 1 Expression and Immune-related Adverse Events in Non-small-cell Lung Cancer Patients Treated with Pembrolizumab

**DOI:** 10.31662/jmaj.2019-0005

**Published:** 2019-11-08

**Authors:** Jun Sugisaka, Yukihiro Toi, Masataka Taguri, Yosuke Kawashima, Tomoiki Aiba, Sachiko Kawana, Ryohei Saito, Mari Aso, Kyoji Tsurumi, Kana Suzuki, Hisashi Shimizu, Hirotaka Ono, Yutaka Domeki, Keisuke Terayama, Atsushi Nakamura, Shinsuke Yamanda, Yuichiro Kimura, Yoshihiro Honda, Shunichi Sugawara

**Affiliations:** 1 Department of Pulmonary Medicine, Sendai Kousei Hospital, Sendai, Japan; 2 Department of Data Science, Yokohama City University school of Data Science, Yokohama, Japan

**Keywords:** Pembrolizumab, immune-related adverse events, immune checkpoint inhibitor, non-small-cell lung cancer, programmed death ligand 1

## Abstract

**Introduction:**

Immune checkpoint inhibitors (ICIs) can lead to immune-related adverse events (irAEs). A correlation between the development of irAEs and efficacy has been suggested; however, it is unclear whether there is a relationship between programmed death ligand 1 (PD-L1) expression and the development of these events.

**Methods:**

We performed a retrospective study of advanced or metastatic non-small cell lung cancer (NSCLC) patients who were treated with pembrolizumab monotherapy at our institution between May 2015 and April 2018 (n = 44). Patients were categorized into two groups, specifically those with irAEs (irAE group) or without (non-irAE group), and we evaluated the objective response rate (ORR), disease control rate (DCR), and progression-free survival (PFS). Predictors of irAEs were examined by multivariate analysis.

**Results:**

irAEs of any grade occurred in 31 (70.5%) patients. The median PFS was 10.9 months in the irAE group versus 3.7 months in the non-irAE group (*P* < 0.001). ORR and DCR were also higher in the irAE group than in the non-irAE group. Furthermore, high PD-L1 expression (≥50%) was a predictive factor of irAE based on logistic regression (*P* = 0.004).

**Conclusions:**

In patients with advanced NSCLC treated with pembrolizumab monotherapy, ORR, DCR, and PFS were significantly better in the irAE group than in the non-irAE group. High PD-L1 expression, at the time of pretreatment, was identified as an independent predictor of irAE development. We believe that more careful management of irAEs for individuals with high PD-L1 expression is needed to improve clinical benefits. Further, PD-L1 expression might be useful for ICI risk management.

## Introduction

Lung cancer, a leading cause of cancer-related death worldwide ^(1)^, is classified into two primary categories, namely small-cell lung carcinoma and non-small-cell lung carcinoma (NSCLC). NSCLC is more common, accounting for approximately 85% of all lung cancers. It can be further divided into two major histological subtypes, specifically adenocarcinoma and squamous cell carcinoma.

Immune checkpoint inhibitors (ICIs) have become standard treatment options for many advanced malignancies. Currently, two anti-programmed cell death-1 (PD-1) inhibitors and two anti-programmed cell death ligand-1 (PD-L1) inhibitors have been approved for treating NSCLC; the former includes nivolumab and pembrolizumab, and the latter are atezolizumab and durvalumab. Based on the clinical trials such as KEYNOTE-189 ^(2)^ and KEYNOTE-407 ^(3)^, pembrolizumab is added to chemotherapy regardless of PD-L1 expression. Pembrolizumab is an engineered, humanized IgG4 antibody ^(4)^, and its target is the programmed cell death protein-1 (PD-1) pathway ^(4), (5)^. PD-1 is an inhibitory co-receptor, primarily expressed on the surface of activated T cells. It binds PD-L1 or PD-ligand 2 (PD-L2) to modulate T cell effector functions including proliferation ^(6)^, cytokine production ^(7)^, and survival ^(8)^.

ICIs can lead to immune-related adverse events (irAEs). Their precise pathophysiology is unknown; however, according to translational trials, T-cell, antibody, and cytokine responses might be involved ^(9)^. We previously reported a correlation between irAEs and efficacy ^(10)^, which was corroborated by similar reports ^(11), (12), (13), (14), (15)^. Despite this knowledge, it is unclear whether there is the relationship between PD-L1 expression and irAEs’ development; to our knowledge, this has not been addressed in a clinical setting. Therefore, we investigated this relationship using NSCLC patients treated with pembrolizumab monotherapy.

## Materials and Methods

This was a retrospective, cohort study of NSCLC patients treated with pembrolizumab monotherapy at Sendai Kousei Hospital (Miyagi, Japan) between May 2015 and March 2018. Patients were selected based on the following criteria: (1) pathologically proven Stage IIIB–IV NSCLC, based on the tumor-node-metastasis staging system (seventh edition) ^(16)^, which includes post-operative recurrence; (2) treatment with pembrolizumab monotherapy; (3) patients who could be evaluated after 8–9 weeks of treatment. We collected the following data: baseline characteristics, best response to pembrolizumab, according to the Response Evaluation Criteria in Solid Tumors (RECIST) version 1.1 criteria, and irAEs according to Common Terminology Criteria for Adverse Events, version 4.0. PD-L1 immunohistochemistry (IHC), using 22C3 pharmDx (Dako North America; Carpinteria, CA) was carried out as a companion diagnostic for the use of pembrolizumab. A tumor proportion score of 50% or more was defined as high PD-L1 expression; a score lower than 50% was defined as low expression. The study protocol was approved by the institutional review board of Sendai Kousei Hospital (IRB no. 30-35). The need to obtain informed consent was waived because the data were analyzed anonymously.

We created a Frontline Immunotherapy Team (FIT), which included specialists from different backgrounds, such as a clinical nurse specialist who interviewed and determined the presence or absence of irAEs before the doctors’ examination. The team carefully observed and documented irAEs.

The patients were categorized into two groups, those with (irAE group) or without (non-irAE group) irAEs, and we evaluated the objective response rate (ORR), disease control rate (DCR), and progression-free survival (PFS). Predictors of irAEs were examined by multivariate analysis.

### Assessment

Objective tumor response to pembrolizumab was confirmed by computed tomography every 8–9 weeks, and this was determined in accordance with the Response Evaluation Criteria in Solid Tumors (RECIST) guidelines, version 1.1 ^(17)^. Two pulmonary physicians (an attending physician and an investigator) measured objective tumor response. The attending physician and a nurse specialist conducted a physical examination, assessed patients for irAEs every three weeks throughout the treatment course, and recorded the results.

According to previous studies ^(11), (18), (19), (20), (21)^, irAEs were defined as adverse events, with potential immunological bases, requiring potential intervention with immunosuppressive or endocrine therapy, such as skin reactions, endocrine, gastrointestinal, hepatic, neurological, and pulmonary irAEs, among others.

### Statistical analyses

Age was analyzed using the Mann-Whitney’s *U* test. Dichotomous variables were analyzed using the Fisher’s exact test. Time to events was estimated, using the Kaplan–Meier method, and compared by the log-rank test. Logistic regression analysis was used for multivariate analysis. The hazard ratio was estimated using the Cox proportional hazards model. The cut-off date for the survival analysis was April 30, 2018.

All statistical analyses were performed with EZR (Saitama Medical center, Jichi Medical University, Saitama, Japan), a graphical user interface for R (R foundation for Statistical Computing, Vienna, Austria). More precisely, it is a modified version of R commander designed to add statistical functions frequently used in biostatistics ^(22)^. *P* < 0.05, based on two-tailed tests, was considered significant.

## Results

### Patient characteristics

The study enrolled 44 patients with NSCLC treated with pembrolizumab monotherapy at Sendai Kousei Hospital (Miyagi, Japan) between May 2015 and April 2018. The characteristics and comparisons among patients are summarized in Table 1. The study included 31 men and 13 women, with a median age of 68 (range, 31–83) years. Two patients were treated with pembrolizumab in the clinical trial, in one as primary therapy and in the other as secondary therapy.

**Table 1.** Baseline Characteristics of the Study Population (n = 44).

Characteristics		Value^a^, n (%)
Age, years, median [range]		68 [31–83]
Sex (male)		31 (70.5%)
ECOG PS at time of pembrolizumab monotherapy^b^		
	0	31 (70.5%)
	1	10 (22.7%)
	2	3 (6.8%)
Smoking status		
	Current smoker or ever smoked	35 (79.5%)
	Never smoked	9 (20.4%)
Pathological subtype		
	Squamous cell carcinoma	17 (38.6%)
	Non-squamous NSCLC	27 (61.4%)
PD-L1 expression		
	≥ 50% (high)	27 (61.4%)
	1–49% (low)	17 (38.6%)
EGFR status in non-squamous NSCLC		
	Mutant	3 (6.8%)
Number of prior chemotherapy regimens		
	0	22 (50%)
	1	13 (29.5%)
	≥ 2	9 (20.5%)
Development of irAEs		31 (70.5%)
Development of severe irAEs		10 (22.7%)
Onset of irAEs, weeks, median [range]		3.3 [0–28]

ECOG, Eastern Cooperative Oncology Group; irAE, immune related adverse event; PS, performance-status; NSCLC, non-small-cell lung cancer; PD-L1, programmed death ligand-1; EGFR, epidermal growth factor receptor; n, number ^a^Values in the table are presented as the median with the range given in square brackets or as a number with the percentage in parentheses. ^b^Eastern Cooperative Oncology Group performance status (ECOG PS) scores range from 0 to 4, with high numbers indicating high disability.

The median PFS was 5.9 months (range 3.9–12.4 months). Of the 44 patients, 18 (40.9%) achieved partial response and 19 (43.1%) achieved stable disease as their best response. Seven (15.9%) experienced disease progression with no benefit from pembrolizumab. None of the patients showed complete response. irAEs of any grade occurred in 31 (70.5%) and severe irAEs (grade ≥ 3) occurred in 10 (22.7%) patients. Twenty two (71.0%) of 31 patients developed irAEs within eight weeks from the start of pembrolizumab monotherapy. Severe irAEs (grade ≥ 3) occurred in eight (29.6%) of 27 patients with high PD-L1 expression and in two (11.8%) of 17 patients with low PD-L1 expression (*P *= 0.27).

Table 2 provides irAE categories. Of the 44 patients treated with pembrolizumab monotherapy, 31 developed irAEs, 18 (40.9%) had a skin reaction, 10 (22.7%) developed pneumonitis, eight (18.2%) had an infusion reaction, four (9.1%) contracted hepatitis, four (9.1%) experienced thyroid dysfunction, two (4.5%) developed microhematuria, one (2.3%) had myocarditis, one (2.3%) presented with pancreatitis, one (2.3%) developed enteritis, and one (2.3%) experienced eye symptoms. Pembrolizumab monotherapy had to be discontinued in 10 patients because of pneumonitis, one due to hepatitis and myocarditis, one because of pancreatitis, and one due to a skin reaction. Systemic steroids were used to treat 14 patients (45.2%). Seven patients fully recovered from irAEs and were able to stop systemic steroids. Among these, one patient was able to resume pemblolizumab treatment without disease progression. Six patients continued systemic steroids. One patient died of pneumonitis. The median onset of irAEs was 3.3 (range, 0–28) weeks.

**Table 2.** Categorization of irAEs in Patient Cohort.

	n (%)	Grade of irAEs, n 1/2/3/4/5
Skin reaction	18 (40.9%)	14/1/3/0/0
Pneumonitis	10 (22.7%)	0/5/4/0/1
Infusion reaction	8 (18.2%)	5/3/0/0/0
Hepatitis	4 (9.1%)	2/0/1/1/0
Thyroid dysfunction	4 (9.1%)	4/0/0/0/0
Microhematuria	2 (4.5%)	2/0/0/0/0
Myocarditis	1 (2.3%)	0/0/0/1/0
Pancreatitis	1 (2.3%)	0/0/1/0/0
Enteritis	1 (2.3%)	0/1/0/0/0
Eye symptom	1 (2.3%)	1/0/0/0/0
Myositis/peripheral neuropathy	0 (0%)	0/0/0/0/0

irAE, immune-related adverse event; n, number

### Association between irAE and clinical outcome

**Figure 1.**
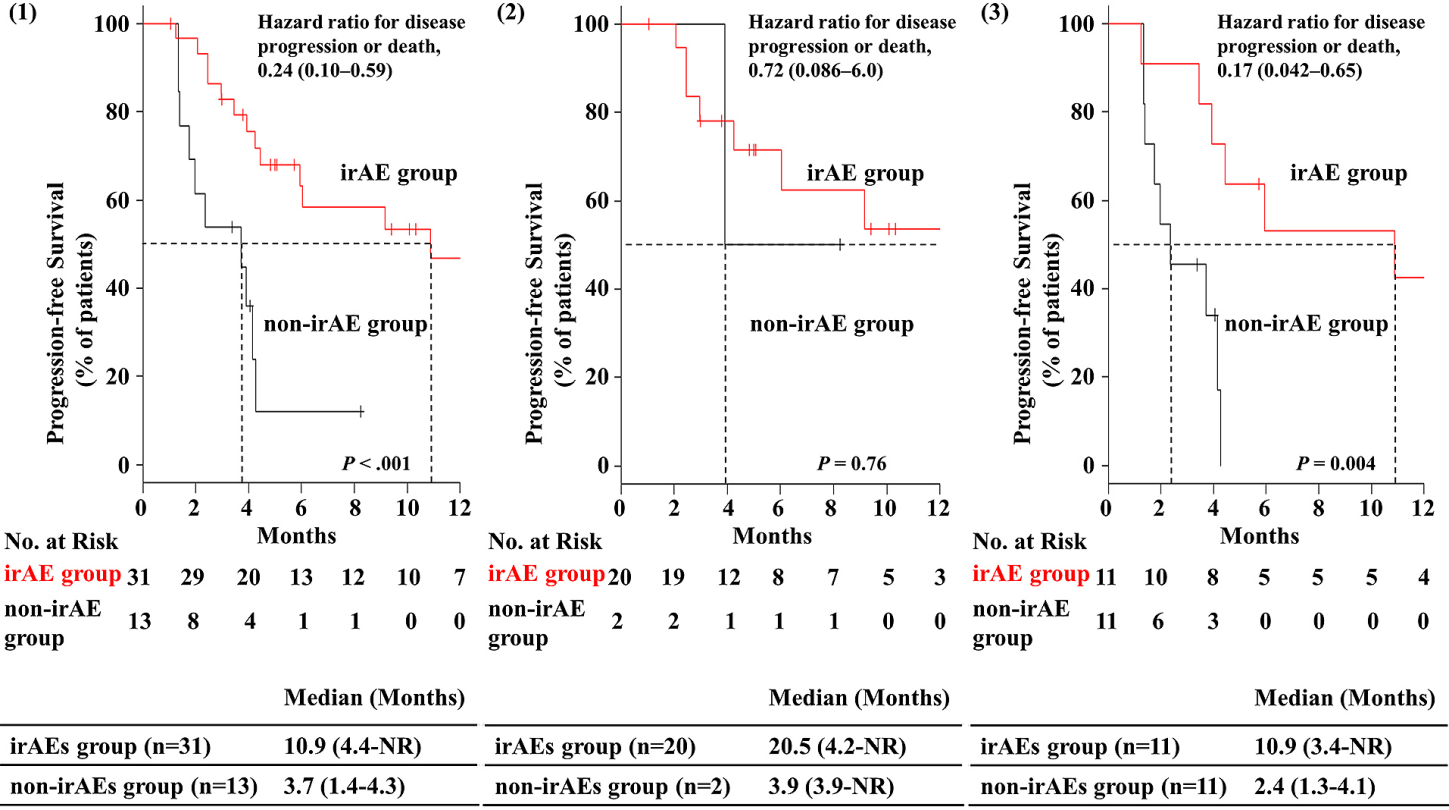
Progression-free survival in NSCLC patients, with or without immune-related adverse events, based on (1) all patients (n = 44), (2) patients treated with pembrolizumab as the primary therapy (n = 22), and (3) patients treated with pembrolizumab as secondary or more therapy (n = 22). Ticks indicate patients whose data were censored on April 30, 2018. irAE, immune related adverse event; NR, not reached.

**Figure 2.**
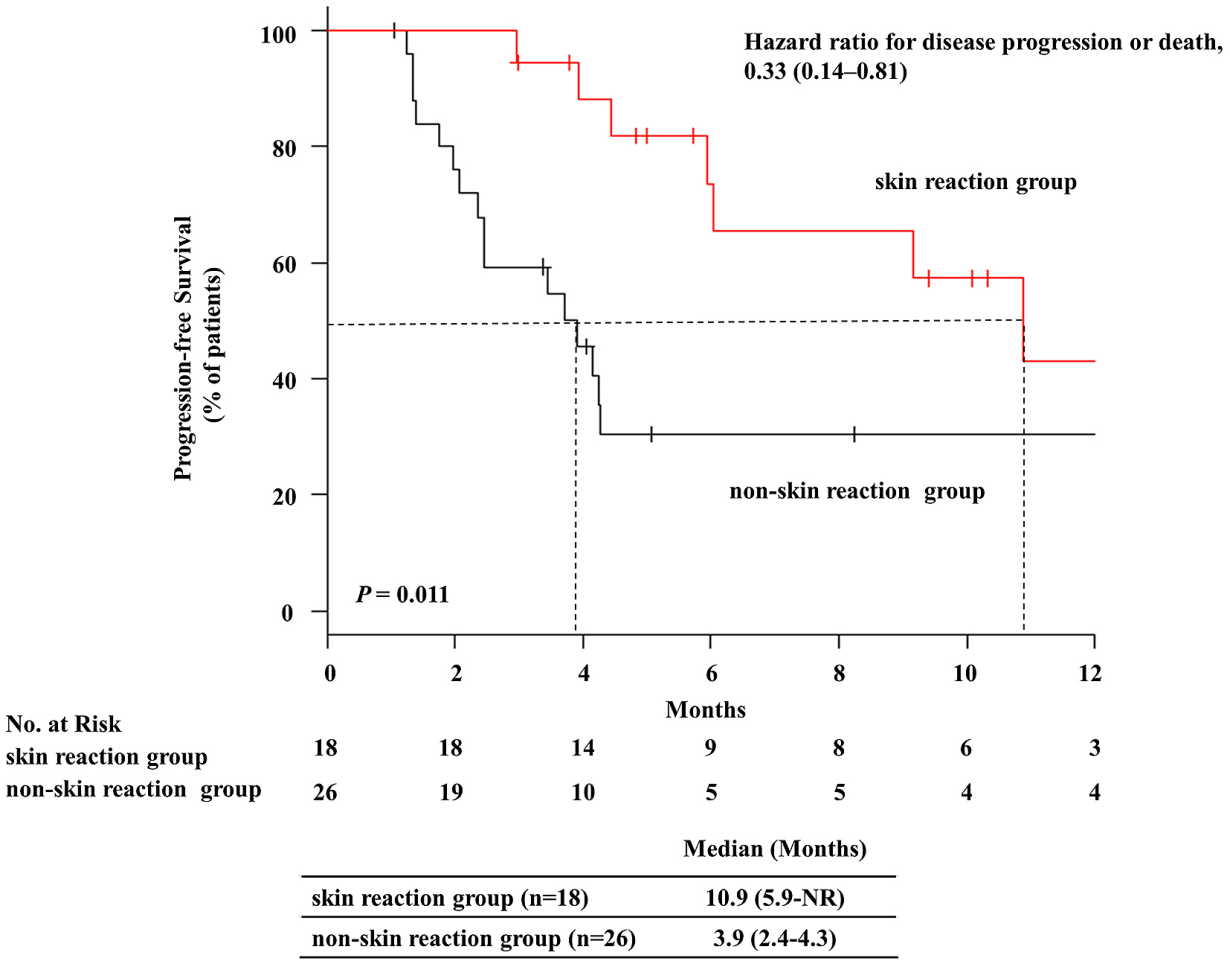
Progression-free survival in NSCLC patients with or without skin reactions. Ticks indicate patients whose data were censored on April 30, 2018. NR, not reached.

Table 3 provides a summary of****characteristics of and comparisons between patients with irAEs (irAE group) and those without (non-irAE group). The ORR was significantly higher in the irAE group than in the non-irAE group (ORR: 51.6% *vs* 15.4%; *P =* 0.043). The DCR was also significantly higher in the irAE group than in the non-irAE group (96.8% *vs* 53.8%; *P* = 0.001). The median PFS was 10.9 months [95% confidence interval (CI): 4.4–not reached] in the irAE group and 3.7 months [95% CI: 1.4–4.3] in the non-irAE group. PFS was remarkably and significantly better in the irAE group than in the non-irAE group (*P <* 0.001). The hazard ratio for disease progression or death was 0.24 (95% CI: 0.10–0.59; Figure 1 (1)). The Kaplan–Meier curves for PFS are shown in Figure 1. Among them, a significant correlation between skin reaction and therapeutic effect (PFS) was observed (*P* = 0.011). The hazard ratio for disease progression or death was 0.33 (95% CI: 0.14–0.81; Figure 2). The median PFS was 10.9 months [95% CI: 5.9–not reached] in the skin reaction group and 3.9 months [95% CI: 2.4–4.3] in the non-skin reaction group. The ORR was significantly higher in the skin reaction group than in the non-skin reaction group (61.1% *vs* 26.9%; *P* = 0.032). Moreover, the DCR was significantly higher in the skin reaction group than in the non-skin reaction group (100% *vs* 73.1%; *P* = 0.031).

**Table 3.** Characteristics of Patients with irAEs (irAE Group) and without irAEs (Non-irAE Group) (n = 44)^a^.

Variables		Univariate analysis			Multivariate analysis		
		irAE group^b^ (n = 31)	non-irAE group^c^ (n = 13)	*P*	Odds rate	95% CI	*P*^h^
Sex (male), n (%)		22 (71.0%)	9 (69.2%)	1.0^f^	-	-	-
Age, years, median [range]		68 [37–83]	71 [31–80]	0.77^g^	-	-	-
Pathological subtype							
	Squamous cell carcinoma, n (%)	13 (41.9%)	4 (30.8%)	0.74^f^	-	-	-
	Non-squamous NSCLC, n (%)	18 (58.0%)	9 (69.2%)				
Smoking (ex, current), n (%)		26 (83.9%)	9 (69.2%)	0.41^f^	-	-	-
ECOG PS 0, n (%)		25 (80.6%)	6 (46.1%)	0.033^f^	4.4	0.85–22.8	0.076
Primary treatment, n (%)		20 (64.5%)	2 (15.3%)	0.007^f^	-	-	-
High PD-L1 expression, n (%)		24 (77.4%)	3 (23.1%)	0.002^f^	11	2.1–54.4	0.004
Best response, n CR/PR/SD/PD		0/16/14/1	0/2/5/6		-	-	-
	Response rate, n (%)^d^	16 (51.6%)	2 (15.4%)	0.043^f^	-	-	-
	Disease control rate, n (%)^e^	30 (96.8%)	7 (53.8%)	0.001^f^	-	-	-

irAE, immune-related adverse event; NSCLC, non-small-cell lung cancer; ECOG PS, Eastern Cooperative Oncology Group Performance Status; PD-L1, programmed death ligand-1; CR, complete response; PR, partial response; SD, stable disease; PD, progression disease; PFS, progression-free survival; CI, confidence interval; n, number; NR, not reached ^a^Values in the table are presented as the median, with the range given in square brackets or as the number with the percentage in parentheses. ^b^IrAE group was defined as patients who developed immune-related adverse events during pembrolizumab monotherapy. ^c^non-irAE group was defined as patients who did not develop immune-related adverse events during pembrolizumab monotherapy. ^d^Response rate was defined as the proportion of patients who achieved complete or partial response based on the modified Response Evaluation Criteria in Solid Tumors version 1.1. ^e^Disease control rate was defined as the proportion of patients who achieved complete or partial response or stable disease based on the modified Response Evaluation Criteria in Solid Tumors Version 1.1. ^f^Results calculated with Fisher’s exact test. ^g^Results calculated with the Mann–Whitney’s *U* test. ^h^Results calculated with logistic regression.

### Predictors of irAE

Based on certain characteristics, univariate analysis identified significantly higher occurrences of high PD-L1 expression, primary therapy, and performance status (PS) 0 in the irAE group, compared to those in the non-irAE group. High PD-L1 expression and PS 0 were then subjected to multivariate analysis. It was revealed that high PD-L1 expression was an independent predictor of irAE development based on logistic regression (*P* = 0.004; Table 3).

Considering only high PD-L1 expression, there were no significant differences in irAE and severe irAE development between primary therapy and second-line or further therapy (Table 4). Similarly, considering only previously-treated patients, there was no significant difference in the development of irAEs and severe irAEs between high and low PD-L1 expression groups (Table 5).

**Table 4.** Comparison of the Development of irAEs and Severe irAEs^a^ in Patients with High PD-L1 Expression Based on Primary Therapy and Secondary Therapy or More (n = 27)^b^.

	Primary therapy (n = 22)	Second therapy or more (n = 5)	*P*^c^
irAEs, n (%)	20 (90.9%)	4 (80.0%)	0.47
Severe irAEs, n (%)	6 (27.3%)	2 (40.0%)	0.62

irAE, immune-related adverse event; PD-L1, programmed death ligand-1 ^a^Severe irAEs indicate irAEs of grade 3 or higher. ^b^Values in the table are presented as the number with the percentage in parentheses. ^c^Results calculated with Fisher’s exact test.

**Table 5.** Comparison of the Development of irAEs and Severe irAEs^a^ in Previously-treated Patients Based on High and Low PD-L1 Expression (n = 22)^b^.

	High PD-L1 expression (n = 5)	Low PD-L1 expression (n = 17)	*P*^c^
irAEs, n (%)	4 (80.0%)	7 (41.2%)	0.31
Severe irAEs, n (%)	2 (40.0%)	2 (11.7%)	0.21

PD-L1, programmed death ligand-1; irAE, immune-related adverse event ^a^Severe irAEs indicate irAEs of grade 3 or more. ^b^Values in the table are presented as the number with the percentage in parentheses. ^c^Results calculated with Fisher’s exact test.

## Discussion

It was previously unclear whether there is a relationship between PD-L1 expression and the development of irAEs, and this has not been addressed in a clinical setting. This study’s purpose was to investigate the relationship between PD-L1 expression and irAEs in NSCLC patients treated with pembrolizumab monotherapy. In this study, there was a significant relationship between PD-L1 expression and irAEs.

In this study, high PD-L1 expression, primary therapy, and performance status (PS) 0 were related to irAE development based on univariate analysis. In Japan, for primary therapy, pembrolizumab monotherapy was used only in patients with high PD-L1 expression over the period of this study; therefore, primary therapy was strongly related to high PD-L1 expression. Therefore, we did not include primary therapy in the multivariate analysis. Based on the multivariate analysis results, there was a significant relationship between PD-L1 expression and irAEs.

In the KEYNOTE-001 trial ^(4)^, patients with high PD-L1 expression were more likely to receive clinical benefits. A global phase 2/3 KEYNOTE-010 study ^(19)^ including NSCLC patients with PD-L1-positive disease with metastases who previously received treatments, showed that the group treated with pembrolizumab exhibited better ORR, median overall survival (OS), and PFS compared to the group treated with docetaxel.

In the KEYNOTE-024 trial ^(20)^, a global phase 3 trial, patients with previously-untreated, advanced NSCLC with high PD-L1 expression (≥50%) experienced longer PFS and OS after pembrolizumab monotherapy compared to those with platinum-based chemotherapy. Furthermore, pembrolizumab might improve or maintain health-related quality of life compared to chemotherapy ^(23)^. In the KEYNOTE-010 trial ^(19)^ and the KEYNOTE-024 trial ^(20)^, irAEs were observed in 19%–29% of patients and severe irAEs occurred at a rate of less than 10%.

Previously, a significant relationship between PD-L1 expression and irAE development was not found in a clinical trial ^(15)^. Moreover, in the BIRCH trial ^(24)^, which studied the effects of atezolizumab, there was no significant correlation between high PD-L1 expression and the development of irAEs. However, in this study, there was a significant relationship between PD-L1 expression and irAEs. The reason is unclear, but it may have been differences among these clinical trials and the actual clinics. Considering the associated mechanism, irAEs could be likely to occur in patients with high PD-L1 expression after ICI treatments. PD-1 and PD-1 inhibitors were also suggested to be important for regulating the humoral immune response. One study reported high PD-1 expression in activated B cells ^(25)^, and this may be modulated by T-cell-dependent and T-cell-independent mechanisms ^(26), (27), (28)^. T cells enhance the treatment effect of PD-1 antibodies, which might induce auto-antibodies via B cells and promote irAE development.

In the present study, there was no significant correlation between high PD-L1 expression and severe irAE development (grade ≥ 3; *P *= 0.27); however, more severe irAEs were observed in patients with high PD-L1, compared to those in individuals with low expression (30.0% *vs* 11.8%). This could be because of a small number of cases.

In this study, irAEs and severe irAEs were observed more frequently compared to the reported frequencies in large clinical trials ^(19), (20)^. However, this might be because our FIT observed irAEs very carefully. For example, conditions such as skin reactions could be easily overlooked without careful examination.

As observed in several other studies ^(10), (11), (12), (13), (14), (15)^, we found that patients with irAEs had better ORR, DCR, and median PFS than patients without irAEs. Upon comparing several characteristics, high PD-L1 expression at the time of pre-treatment was identified as an independent predictor of irAE development. Thus, for patients with high PD-L1 expression, attention should be paid to forthcoming irAEs, and appropriate management of these events could result in clinical benefits. In the irAE group, 70% of irAEs occurred within eight weeks. As in a previous study ^(10)^, most irAEs occurred early. Thus, we propose that longer pembrolizumab treatment does not lead to more irAEs.

In clinical trials on adding ICI to chemotherapy ^(2), (3)^, clinical benefits can be expected regardless of PD-L1 expression. In the future, when we use ICI for NSCLC, the measurement of PD-L1 expression might become unnecessary. In this study, there was a significant relationship between PD-L1 expression and irAEs; thus, PD-L1 expression might be a useful marker for risk management with ICIs. Although future studies are necessary, this marker might also be useful for risk management with respect to the addition of ICIs to chemotherapy.

There were some limitations to this study. First, it was retrospective, performed at a single institution, and had a small sample size. Second, there could have been some observer bias. However, to the best of our knowledge, we believe that this is the first study to focus on a relationship between PD-L1 expression and f irAE development and report that high PD-L1 expression might be related to irAE development.

In conclusion, in patients with advanced NSCLC treated with pembrolizumab monotherapy, ORR, DCR, and PFS were significantly better in the irAE group than in the non-irAE group. Moreover, high PD-L1 expression at the time of pre-treatment was identified as an independent predictor of irAE development. We suggest that more careful management of irAEs in those with high PD-L1 expression, compared to that for individuals with low PD-L1 expression, is needed to achieve better clinical benefits. Moreover, PD-L1 expression might be a useful marker for the risk management of ICI.

## Article Information

### Conflicts of Interest

Dr Sugawara received honoraria from MSD, Ono Pharmaceutical, Bristol-Myers Squibb, AstraZeneca, Chugai Pharma, Nippon Boehringer Ingelheim, Pfizer, Taiho Pharmaceutical, Eli Lilly and Company, Novartis, and Kyowa Hakko Kirin; Dr Toi received honoraria from MSD, Ono Pharmaceutical, and Bristol-Myers Squibb; Dr Saito received honoraria from Bristol-Myers Squib; Dr Domeki received honoraria from Ono Pharmaceutical and Bristol-Myers Squibb; Dr Nakamura received honoraria from MSD.

### Acknowledgement

The authors thank Chieko Hattori, chief nurse of Sendai Kousei Hospital, and all members of the Frontline Immunotherapy Team (FIT) at Sendai Kousei Hospital.

### Author Contributions

Conception/design: Jun Sugisaka, Shunichi Sugawara, Yukihiro Toi

Data analysis and interpretation: Jun Sugisaka, Shunichi Sugawara, Yukihiro Toi, Masataka Taguri, Yosuke Kawashima, Tomoiki Aiba, Sachiko Kawana, Ryohei Saito, Mari Aso, Kyoji Tsurumi, Kana Suzuki, Hisashi Shimizu, Hirotaka Ono, Yutaka Domeki, Keisuke Terayama, Atsushi Nakamura, Shinsuke Yamanda, Yuichiro Kimura, Yoshihiro Honda

Manuscript writing: Jun Sugisaka

Final approval of manuscript: Jun Sugisaka, Shunichi Sugawara, Yukihiro Toi, Masataka Taguri, Yosuke Kawashima, Tomoiki Aiba, Sachiko Kawana, Ryohei Saito, Mari Aso, Kyoji Tsurumi, Kana Suzuki, Hisashi Shimizu, Hirotaka Ono, Yutaka Domeki, Keisuke Terayama, Atsushi Nakamura, Shinsuke Yamanda, Yuichiro Kimura, Yoshihiro Honda

### Approval by Institutional Review Board (IRB)

IRB no. 30-35

The institutional review board of Sendai Kousei Hospital
